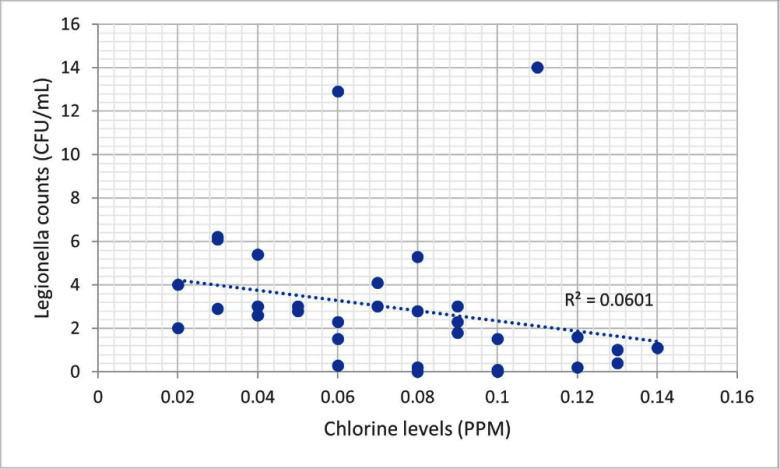# Chlorine levels and a Legionella outbreak

**DOI:** 10.1017/ash.2024.341

**Published:** 2024-09-16

**Authors:** Sean Wu, Jyoti Somani, Hwang Ching Chan, Nazira Fauzi

**Affiliations:** National University Hospital (Singapore)

## Abstract

**Background:** Legionella, first identified in the 1970s, is increasingly recognized as an opportunistic pathogen in healthcare facilities.1 The National University Hospital (NUH) is a large quaternary level academic hospital with 1,200 beds located in equatorial Singapore. Its first building, completed in 1985, still serves patients today. In 2022, the infection prevention team (IPT) was informed of two cases of nosocomial legionella, which sparked the start of an extensive investigation consisting of water quality testing of multiple sources, case finding, and formation of a water management committee. **Methods:** 250mL water samples were collected and cultured by an external vendor using direct membrane filtration followed by plating on selective agar according to ISO 11731 standards. Bacterial colonies were then identified, quantified and speciated. At the same time, NUH’s facility management measured chlorine levels using a portable colorimeter. **Results:** In total, we cultured 34 samples taken from sinks, shower heads, potable water dispensers. 91.2% of samples were positive for legionella. Of the 91.2%, 35.3% of sites grew Legionella pneumophila serogroup 1 while 79.4% of sites grew Legionella pneumophila serogroup 2-15. We attributed our high rates of legionella positivity to an aging plumbing system and Singapore’s high humidity and temperatures. In addition, the maximal temperature of our hot water is only 48-50 °C. Although chlorine levels were generally low, they were still within the local recommendation of less than 2ppm (Singapore does not have guidance on minimum chlorine levels). We found no statistically significant correlation between the number of legionella colony forming units (CFUs) and chlorine levels (ranging between 0.02 to 0.14ppm). This supports the United States Environmental Protection Agency’s recommendation, as well as the findings from in vitro and in vivo studies, for a minimal chlorine of 0.2 PPM at the taps for acute care hospitals.2,3However, these levels may be inadequate in the presence of acanthamoeba or a high biofilm load within water systems.4,5 **Conclusion:** Hospital water management programs should require a minimal level of chlorine at hospital taps and at levels above those recommended by public water systems, in order to control legionella growth. In addition, the formation of a hospital water management committee is essential to improve hospital water quality and put mitigation measures in place. References 1. Phin, N. et al. Epidemiology and clinical management of Legionnaires’ disease. Lancet Infect. Dis. 14, 1011–1021 (2014). 2. Marchesi, I. et al. Monochloramine and chlorine dioxide for controlling Legionella pneumophila contamination: biocide levels and disinfection by-product formation in hospital water networks. J. Water Health11, 738–747 (2013). 3. Cervero-Aragó, S., Rodríguez-Martínez, S., Puertas-Bennasar, A. & Araujo, R. M. Effect of Common Drinking Water Disinfectants, Chlorine and Heat, on Free Legionella and AmoebaeAssociated Legionella. PloS One 10, e0134726 (2015). 4. Kessler, M. A., Osman, F., Marx, J., Pop-Vicas, A. & Safdar, N. Hospital-acquired Legionella pneumonia outbreak at an academic medical center: Lessons learned. Am. J. Infect. Control 49, 1014–1020 (2021).

**Figure:**